# Transitions from child and adolescent to adult mental health services for eating disorders: an in-depth systematic review and development of a transition framework

**DOI:** 10.1186/s40337-024-00984-3

**Published:** 2024-03-07

**Authors:** Anya Ragnhildstveit, Nandita Tuteja, Paul Seli, Leo Smart, Naz Uzun, Lisa C. Bass, Alyssa C. Miranda, Tamsin J. Ford, Sharon A. S. Neufeld

**Affiliations:** 1 Department of Psychiatry, University of Cambridge, Cambridge, England, UK; 2 Department of Neurobiology and Behavior, University of California, Irvine, Irvine, CA USA; 3 Department of Psychology and Neuroscience, Duke University, Durham, NC USA; 4 Neuroscience Program, Bates College, Lewiston, ME USA; 5 Department of Psychology, University of Manchester, Manchester, England, UK; 6 Neuroscience Interdepartmental Program, University of California, Los Angeles, Los Angeles, CA USA; 7 Consciousness and Transformative Studies, National University, San Diego, CA USA

**Keywords:** Eating disorder, Anorexia nervosa, Bulimia nervosa, Binge eating disorder, Mental health services, Age transitions, Systematic review

## Abstract

**Background:**

Eating disorders (EDs) peak in mid-to-late adolescence and often persist into adulthood. Given their early onset and chronicity, many patients transition from child and adolescent mental health services (CAMHS) to adult mental health services (AMHS) for ongoing, speciality ED care. This transition typically occurs at 18 years of age, when important biological, psychosocial, and vocational changes take place. Thus, smooth and effective transitions are paramount for ensuring service continuity, as well as reducing the risk of ED relapse and premature death. Here, we synthesized evidence on transitions from CAMHS to AMHS for young people with EDs, aiming to inform future research, clinical practice, and healthcare policy.

**Methods:**

A systematic review of the literature was conducted. This adhered to PRISMA guidelines. PubMed, Embase, and Scopus electronic databases were queried from inception to December 3, 2023. Leveraging the PICOS framework, study eligibility was evaluated in the qualitative synthesis. Data regarding methodology, analytic approach, and associated outcomes were then extracted. The quality of evidence was examined using critical appraisal tools. Finally, concept mapping was applied to organize findings into a transition framework.

**Results:**

The search returned 76 articles. Of these, 14 were included in the final review. Articles were grouped into ‘qualitative’ (*n* = 10), ‘cross-sectional’ (*n* = 2), and ‘longitudinal cohort’ (*n* = 2) studies based on research design. Overall, ED transitions were complex, multifaceted, and challenging for patients, caregivers, and providers alike. This resulted from an interplay of temporal- (e.g., timing of ED onset and transition), stakeholder- (e.g., patient ambivalence towards recovery) and systemic- (e.g., differences between services) related factors. Most studies were of moderate-to-high quality. Findings informed the development of five transition strategies designed to facilitate effective transfers across ED care: Timely talks, Readiness, Inclusion, Preparation, and Synergy (TRIPS).

**Conclusions:**

Transitions from CAMHS to AMHS appear problematic for young people with EDs and other involved stakeholders. The field stands to benefit from TRIPS, an actionable, evidence-based framework that aims to alleviate challenges of transitioning and subsequently improve ED trajectories. As a logical next step, future work should empirically test the TRIPS framework, exploring its predictive utility and clinical value.

**Supplementary Information:**

The online version contains supplementary material available at 10.1186/s40337-024-00984-3.

## Abstract (plain language summary)

Eating disorders often develop in youth and persist into adulthood. Given this, many young people transition from pediatric to adult care for ongoing treatment. This usually occurs at 18 years of age, when important life changes take place, such as leaving home or pursuing higher education. Hence, smooth and effective transitions are critical for mental health. The present review summarized studies investigating transitions from pediatric to adult care for young people with eating disorders, and subsequently developed an evidence-informed transition framework (TRIPS). Based on the 14 studies included in the review, transitions from pediatric to adult care are challenging for young people with eating disorders, as well as for caregivers and providers. This is due to several factors related to the timing of transitions, the types of stakeholders involved, and the differences between care. Looking ahead, the field may benefit from the TRIPS framework that aims to improve transitions and clinical outcomes.

**Supplementary Information:**

The online version contains supplementary material available at 10.1186/s40337-024-00984-3.

## Introduction

Eating disorders (EDs) are highly complex, potentially fatal, psychiatric diseases. They are characterized by abnormal preoccupations with food and eating behaviors, along with body image disturbances [1]. Overtime, the effects of EDs can result in widespread, multi-organ complications, including cardiac and cerebral atrophy [2–4]. As a consequence, EDs have the highest morality rate of any psychiatric disease [5–7]: five-fold that of the general population, according to age and sex [5]. Suicide is also markedly elevated [8], accounting for one in five reported deaths in anorexia nervosa (AN) [9]. Anxious and impulsive traits, common to those with EDs, such as neuroticism and novelty-seeking, may converge to increase suicidal risk [8].

Typically, the age of ED onset peaks in mid-to-late adolescence, depending on clinical presentation, and persists into early, middle, or late adulthood. For instance, AN has a peak age of onset of 15.5 years [10], with a mean illness duration of 10 years [1]. Given the early onset and chronicity of EDs, many young people require ongoing, specialty care as adults, transitioning from child and adolescent mental health services (CAMHS) to adult mental health services (AMHS) for their condition. In high-income countries, this often occurs at 18 years of age, according to service provisions [11, 12]. However, transitions have been widely criticized by experts in the field [13], as their timing coincides with peak ED onset and important life changes, such as structural brain development [14], increased self-autonomy and independence [15], and pursuit of higher education [16]. This can lead to a lack of motivation and readiness for transitioning, leaving young people feeling overwhelmed and unsupported [17].

Moreover, nearly 50% of young people lose contact with services after discharging from CAMHS, falling into the ‘service gap’ [18, 19]. This is commonly due to unsuccessful referrals, failure to meet clinical thresholds for adult care, or refusing to accept ongoing treatment [20]. Thus, transitions are a growing international concern among scientists, clinicians, and policymakers [18, 21–24], with less than 5% of young people who undergo the transition to AMHS experiencing continuity of care, as revealed by the Transition from CAMHS to Adult Mental Health Services (TRACK) study [25]. Smooth and effective transitions  are particularly challenging, given differences in treatment philosophy, approach, and delivery between services [21], as well as unstandardized transition protocols or lack thereof [18].

In light of these issues, transitions are frequently poor and often result in service disengagement, mental health deterioration, and psychosocial impairment [26]. For young people with EDs, this “…can cause delays in commencing or continuing treatment, disruptions to the therapeutic alliance, and even death” [1]. Previously, Wade et al. [27] explored potential solutions to challenges caused by ED transitions, including all-age integrated care. However, this service overhaul is likely premature, given the lack of supporting evidence, with no quantitative studies evaluating this approach to date [27, 28]. As such, we present an updated review on transitions in young people with EDs, aiming to identify optimal strategies for existing binary care models, and to inform future research, clinical practice, and healthcare policy. An actionable, evidence-based transition framework is proposed.

## Methods

A systematic review of the literature was performed, using a targeted search strategy. This adhered to the Preferred Reporting Items for Systematic Reviews and Meta-Analyses (PRISMA) guideline [29]. The review protocol was registered a priori with the Open Science Framework (OSF) [30] (10.17605/OSF.IO/R8TSH).

### Search strategy

PubMed (National Library of Medicine), Embase (Elsevier), and Scopus (Elsevier) electronic databases were queried for relevant articles from inception to December 3, 2023. The full search strategy is detailed in Additional file 1: Table S1. No search restrictions were applied; however, articles were filtered by field (titles), article type (peer-reviewed), and language (English).

### Eligibility criteria

The Population, Intervention, Comparison, Outcomes, and Study design (PICOS) [31] framework was applied to evaluate eligibility in the qualitative synthesis (Additional file 1: Table S2). Inclusion criteria consisted of the following: (1) young people (aged 15–25) diagnosed with an ED of any type, severity, and duration; caregivers of young people diagnosed with an ED; or providers treating young people diagnosed with an ED; (2) transitions from CAMHS to AMHS for an ED, whether impending or completed, in tertiary or community care settings; (3) experiential (attitudes, perspectives, or experiences) or clinical (symptoms, health, function, quality of life, or survival) outcomes related to any step of the transition pathway, including referral, assessment, treatment, or discharge; (4) qualitative, quantitative, or mixed method studies, whether retrospective or prospective; and (5) articles peer-reviewed and published in the English language. In contrast, exclusion criteria consisted of the following: (1) infants, children, or adolescents (aged < 15) and young people not diagnosed with an ED; (2) transitions from CAMHS to AMHS for a non-ED diagnosis; (3) experiential or clinical outcomes not related to any step of the transition pathway; (4) case studies, reviews, editorials, opinion pieces, commentaries, letters to the editor, or studies with inaccessible full texts; and (5) articles not peer-reviewed or published in the English language. Duplicates were removed prior to screening and studies failing to meet full inclusion criteria were excluded from the analysis.

### Article screening

Two independent reviewers (L.S. and N.T.), one with and one without prior content knowledge, screened articles against PICOS criteria, initially evaluating their titles and abstracts. Relevant articles were then selected for full-text screening and assessed for eligibility. Inter-rater agreement was determined using Cohen’s kappa, with disagreements reconciled by a third independent reviewer (A.R.) until a consensus was reached.

### Data extraction

Data from eligible studies were charted into a Microsoft Excel Spreadsheet. Table cells were labeled as ‘Not Applicable’ (N/A) if parameters of interest were missing. To safeguard data, an independent reviewer (L.C.B.) performed quality assurance checks at random. Data extracted from eligible studies included: publication year, study objective, site location, sample size, research design, analytic approach, primary outcomes, and any other pertinent findings. Following extraction, data were qualitatively described, using frequency (count or percentage), central tendency (mean, median, or mode), and variability (range or standard deviation), as applicable via R version 4.2.3 [32]. Pooled statistical analyses, such as meta-regressions, were not performed due to study-observed heterogeneity.

### Quality assessment

To assess the quality of evidence, a critical appraisal of each study was conducted. This considered the extent of possible bias in its design, conduct, and analysis. Three critical appraisal tools were utilized, depending on research design, including the Critical Appraisal Skills Program Checklist for Qualitative Studies [33], the Joanna Briggs Institute Critical Appraisal Checklist for Analytical Cross-Sectional Studies [34], and the Critical Appraisal Skills Program Checklist for Cohort Studies [35]. Using a Microsoft Excel Spreadsheet, responses to signaling questions (detailed in the results tables) were mapped onto proposed bias judgements across several domains. Possible judgements (‘yes’, ‘no’, or ‘unclear’) reflected how adequately signaling questions were addressed. Two independent reviewers (A.R and N.T.) performed critical appraisals, with inter-rater agreement determined using Cohen’s kappa.

### Concept mapping

Applying principles of concept mapping [36], factors reported in the literature (e.g., barriers and facilitators) were compiled, sorted, and mapped into a conceptual pool of determinants of ED transitions, and used to specify a theoretically grounded framework. Preliminary groupings of factors were discussed amongst authors, subsequently refined by consensus, and finally ranked by frequency (i.e., how often they appeared in the literature). Thereafter, dominating factors were translated into five actionable transition strategies.

## Results

**Fig. 1:**
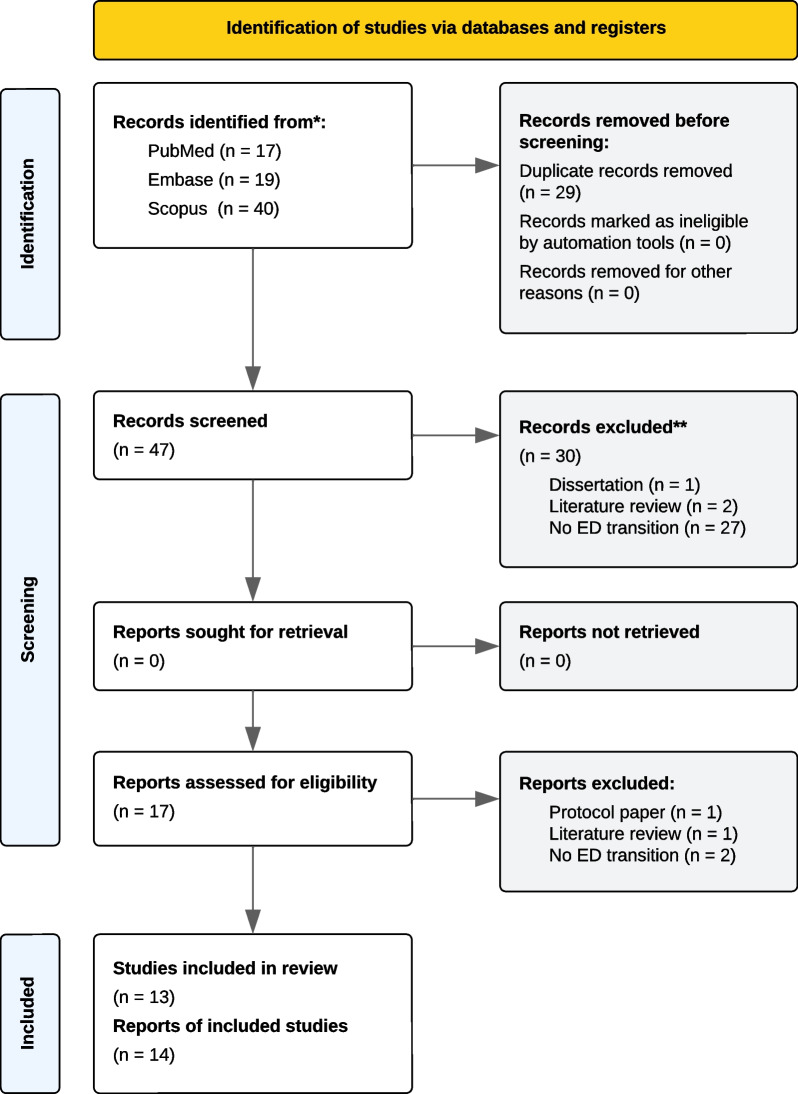
PRISMA flowchart

The PRISMA 2020 flow diagram, describing the search strategy and selection schema, is displayed in Fig. 1. The search returned 76 articles for potential inclusion in the review (PubMed [*n* = 17], Embase [*n* = 19], and Scopus [*n* = 40]). Of these, 47 (62%) unique articles remained following removal of duplicates (38% [*n* = 29]). After title and abstract screening, 30 (64%) articles were excluded for being literature reviews (4% [*n* = 2]), not investigating ED transitions (57% [*n* = 27]), or not being peer reviewed (2% [*n* = 1]). Accordingly, 17 (36%) articles were assessed for eligibility, with 4 (24%) deemed non-eligible for inclusion. One additional article was identified via reference checking. There were no inaccessible full texts. Between independent reviewers who screened articles, there was almost perfect agreement (Cohen’s *k* = 0.94).

The final review comprised 14 articles, with a total of 747 (53.36 [mean] ± 92.58 [std dev.]) participants. The first study was published in 2008 (*n* = 1), the largest number was published between 2020 and 2021 (*n* = 6), and the most recent was published in 2023 (*n* = 3). Regarding location, most studies were conducted in England (36% [*n* = 5]) and Canada (36% [*n* = 5]), followed by Norway (21% [*n* = 3]) and France (7% [*n* = 1]). At the time of data abstraction, articles were grouped into ‘qualitative’, ‘cross-sectional’, and ‘longitudinal cohort’ studies by research design. Ten studies conducted focus groups and/or qualitative interviews (*n* = 153 [15.30 ± 8.12]), two studies distributed prospective surveys at one time point (*n* = 66 [33.00 ± 7.07]), and two studies analyzed data retrospectively (*n* = 528 [264.00 ± 82.02]). Concerning participants, five studies recruited patients (*n* = 559 [111.80 ± 144.90]), one study recruited caregivers (*n* = 12 [12.00 ± 0.00]), four studies recruited providers (*n* = 87 [21.75 ± 12.50]), and four studies recruited more than one type (*n* = 89 [22.25 ± 10.28]).

### Qualitative studies

**Fig. 2:**
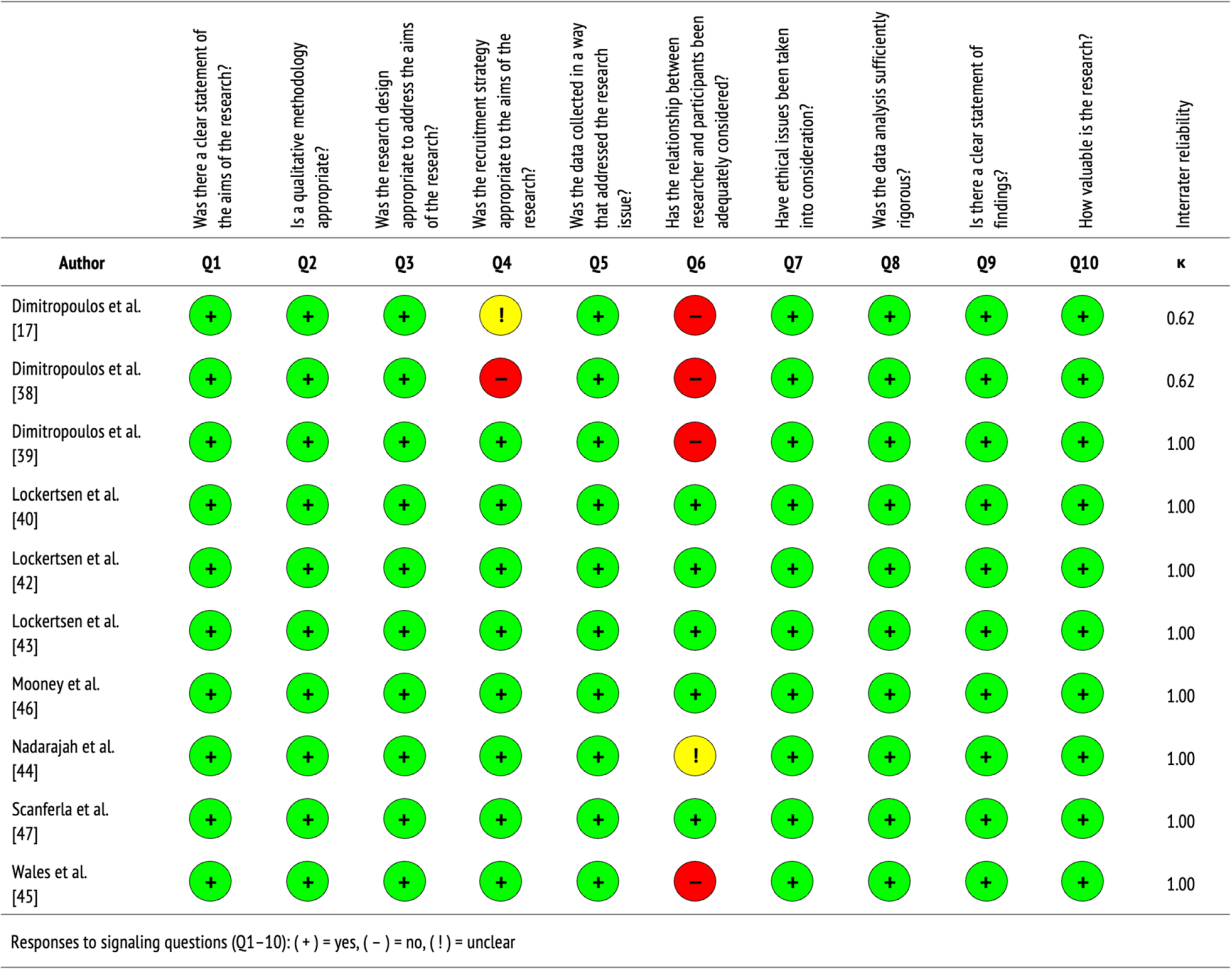
Quality assessment of qualitative studies

See Table 1 for a summary of the qualitative studies included in this review, which are further described below. Figure 2 displays the quality of evidence, specifically the extent to which each study addressed the possibility of bias in its design, conduct, and analysis. Overall, qualitative studies were of moderate-to-high quality, with almost perfect agreement between independent reviewers who performed critical appraisals (Cohen’s *k* = 0.92).

**Table 1:** Characteristics of qualitative studies

Author	Objective	Location	Sample	Study design	Analysis	Key themes
Dimitropoulos et al. [17]	To understand factors that affect transitions from PEDPs to AEDPs in young people with AN	Eating Disorders Program, Toronto General Hospital, Canada	Providers (*n* = 18 +)	In-depth, semi-structured focus groups (2 h. × 2); in-depth, semi-structured interviews (1 h. × 5)	Grounded theory; triangulation	Barriers: illness-related factors, developmental interruption, and decline in parental involvement with related service withdrawal
Dimitropoulos et al. [38]	To understand factors that influence effective transitions from PEDPs to AEDPs for AN	Eating Disorders Program, Toronto General Hospital, Canada	Providers (*n* = 23)	In-depth, semi-structured focus groups (2 h. × 2); in-depth, semi-structured interviews (1 h. × 5)	Grounded theory; triangulation	Factors: readiness (not age), transition-specific interventions for patients and families, and coordinated medical follow up
Dimitropoulos et al. [39]	To identify barriers and facilitators to transitioning from PEDPs to AEDPs in young adults with EDs	Eating Disorders Program, Toronto General Hospital, Canada	Patients (*n* = 15)	In-depth, structured interviews (1 h.)	Grounded theory; triangulation	Barriers: inconsistent transition procedures and systemic barriers to recovery after transitioning to AEDPs; facilitators: better coordination, communication, and collaboration
Lockertsen et al. [40]	To explore how providers experience the transition from CAMHS to AMHS for patients with AN	South-Eastern Norway Regional Health Authority, Norway	Providers (*n* = 8)	Dialectic, multi-step focus group (1.5 h. × 1); in-depth, semi-structured interviews (1.5 h. × 2)	Malterud’s systematic text condensation	Barriers: different treatment cultures, mistrust between services, clinician insecurity, and lack of transfer alliance
Lockertsen et al. [42]	To understand how patients with AN experience the transition from CAMHS to AMHS	South-Eastern Norway Regional Health Authority, Norway	Patients (*n* = 10)	Dialectic, multi-step focus group (1–1.5 h. × 1); in-depth, semi-structured interviews (1–1.5 h. × 5)	Giorgi’s systematic text condensation	Experiences: lack of preparation and related loneliness, not treated uniquely, time to build provider trust, and provider interactions
Lockertsen et al. [43]	To explore how parents experience the transition from CAMHS to AMHS for children with AN	South-Eastern Norway Regional Health Authority, Norway	Parents (*n* = 12)	In-depth, semi-structured interviews (1–1.5 h.)	Giorgi’s systematic text condensation	Barriers: sudden discharge, lack of continuity of care, poor involvement in process, and overwhelming responsibility; facilitators: provider knowledge and professional support
Mooney et al. [46]	To assess the value of educational resources to support young people with AN in transitioning from CAMHS to AMHS	Janeway Children’s Health and Rehabilitation Centre, Canada	Patients(*n* = 6)	In-depth, semi-structured interviews (30 min.)	Thematic analysis	Findings: educational resources as benchmarks for evaluating ED status and tools for connecting with new providers in AMHS
Nadarajah et al. [44]	To identify barriers and facilitators to impending transitions from CAMHS to AMHS for adolescents with EDs	McMaster Children’s Hospital, Canada	Patients, caregivers (*n* = 10)	In-depth, semi-structured interviews (0.5–1 h.)	Summative content analysis	Barriers: re-explaining/re-sharing information, lack of professional support, and late discussions; facilitators: parental involvement and transition coordinators or passport
Scanferla et al. [47]	To capture shared transition experiences from PEDPs to AEDPs among young people with AN and their families	Paris Psychiatry and Neuroscience University Hospital Group, France	Patients, caregivers (*n* = 18)	In-depth, semi-structured interviews	Interpretive phenomenological analysis	Barriers: delayed access to care, adverse effects, and lack of provider support; facilitators: supporting personal life goals and involving caregivers in the transition process
Wales et al. [45]	To understand the transition from CAMHS to AMHS for EDs; and to identify factors that influence this process	National Health Services, England	Patients, caregivers, providers (*n* = 33)	In-depth focus groups (1 h. × 4); in-depth, semi-structured interviews (50 min × 11)	Thematic analysis	Factors: communication, service differences, and transition timing; improved communication, clear expectations, and flexibility may enhance transitions

*AEDPs* adult eating disorder programs, *AMHS* adult mental health services, *AN* anorexia nervosa, *CAMHS* child and adolescent mental health services, *EDs* eating disorders, PEDPs pediatric eating disorder programs

Dimitropoulos et al. [17] were the first to conduct qualitative research on the perspectives of providers, covering the transition of young people from CAMHS to AMHS for AN. Two in-depth, semi-structured focus groups (2 h) were conducted, one at a pediatric ED program (*n* = 8) and one at an adult ED program (*n* = 10). Providers had direct experience with transitioning patients in the last 12 months. In addition, five in-depth, semi-structured qualitative interviews (1 h) were conducted in tertiary and community care settings with providers treating patients who had exited pediatric ED programs, yet who had not accessed adult ED services. Using grounded theory methodology, an approach for generating theory based on systematic data [37], three inter-related themes were identified: (1) AN-related factors, namely denial of illness and ambivalence towards recovery; (2) developmental interruptions in adolescence, such as failure to cultivate autonomy and social connections; and (3) reduced parental involvement in adult ED services, with change in authority and legal power. There was congruency within and across focus groups and qualitative interviews.

In a follow-up study [38] that recruited providers from the same ED programs, investigators sought to identify factors that impact effective transitions for AN. Similarly, two in-depth, semi-structured focus groups (2 h) were conducted, one at a pediatric ED program (*n* = 8) and one at an adult ED program (*n* = 10), along with five in-depth, semi-structured qualitative interviews (1 h). The latter involved professionals outside of these programs (*n* = 5). Their analysis revealed two major themes. First, transitions to AMHS should be determined by patient and family readiness, not by age, as emphasized in the pediatric ED focus group and qualitative interviews. However, this was not identified as problematic in the adult ED focus group. Second, transitions can be improved by family interventions and psychoeducation, patient interventions and self-management skills, and coordinated medical care before or immediately after transitioning from CAMHS. These suggestions were shared in both focus groups and qualitative interviews. Further, all providers agreed that interventions should foster greater patient autonomy and independence.

Findings from both studies motivated a third [39], regarding the experiences of young adults (aged 17–21) with EDs who transitioned from CAMHS to AMHS. Patients were eligible if they met diagnostic criteria for AN or BN and received treatment in a tertiary pediatric ED program within the past two years. In-depth, structured qualitative interviews (1 h) were conducted across two sites (*n* = 15), which were geared towards patients’ retrospective experiences. Three key themes emerged: (1) difficulty navigating care during the transition period, largely due to inconsistent procedures and minimal provider discussions; (2) challenge achieving and maintaining recovery post-transition as a result of systematic barriers, such as accessing care and trained providers, and the intensity of available programs interfering with higher education; and (3) transitions can be improved by increased coordination, communication, and collaboration between services and providers.

As part of a larger, three-study qualitative project, Lockertsen et al. [40] explored how providers experience the transition process for AN. Using snowball sampling, providers (*n* = 8) were recruited from in- and out-patient facilities who had experience treating and/or coordinating treatment for AN patients, plus transitioning them across care. One dialectic, multi-step focus group (1.5 h) and two in-depth, semi-structured qualitative interviews (1.5 h) were conducted. Leveraging systematic text condensation, a method for analyzing cross-case qualitative data [41], their analysis revealed four primary barriers to transitioning. This included (1) differences in treatment cultures between services, with respect to family involvement and patient responsibility; (2) a lack of mutual understanding between services, regarding administrative systems and treatment ideologies and approaches; (3) feelings of ineptitude and insecurity and poor self-confidence among providers, negatively impacting patients and their transition experience; and (4) a lack of trust between services—across systems, providers, and patients—and focus on building transfer alliance. Additionally, providers expressed that parents and patients were not adequately prepared for the transition process.

These results extended to another study [42] that examined patients’ lived experiences transitioning from CAMHS to AMHS. Ten patients with AN, previously treated at in-patient units (*n* = 6) or specialized ED programs (*n* = 3), were recruited through providers and ED support groups. They partook in one dialectic, multi-step focus group (1–1.5 h) and five in-depth, semi-structured qualitative interviews (1–1.5 h). Four inter-related themes were identified: (1) a lack of preparedness for transitioning and related loneliness and stress; (2) time needed to establish relationships with providers, deemed essential for developing trust, mutual understanding, and motivation for treatment; (3) poor acknowledgement of patient individuality and self-sufficiency, with transitions based on age versus developmental stage and readiness; and (4) dependency on systems and provider impressions, causing feelings of powerlessness and hopelessness.

As the final study in this project, Lockertsen et al. [43] assessed how parents experience the transition process for adolescents with AN. Through snowball sampling, they recruited mothers (*n* = 9) and fathers (*n* = 3) via therapist referrals and study advertisements. All parents had a child diagnosed with AN who transitioned to AMHS at 18 years of age. In-depth, semi-structured qualitative interviews were conducted (1–1.5 h) in settings chosen by parents. Using systematic text condensation, their analysis revealed six themes: (1) sudden discharge from CAMHS that was determined by age, rather than by process; (2) lack of continuity between services, followed by mental health deterioration during waiting periods; (3) poor involvement and access to information, resulting in psychological distress; (4) overwhelming and complex responsibility, making the timing of transitions challenging; (5) provider incompetency and ignorance of both patients’ and parents’ ED knowledge and understanding, contributing to many unnecessary transitions; and (6) a lack of professional support from mental health services.

Taking a prospective approach, one study [44] investigated impending transitions for adolescents (*n* = 5) and their caregivers (*n* = 5). Patients were eligible if they met diagnostic criteria for an ED, as determined by a psychologist or physician; were actively being treated in CAMHS, specifically in a tertiary ED program; and were aged 17–18, waiting to undergo the transition to AMHS. In-depth, semi-structured qualitative interviews (0.5–1 h) were conducted. Using inductive reasoning, analyses showed that stakeholders largely had a limited understanding of the transition process. This applied more so to caregivers than adolescents. Several barriers to transitioning were described, including late timing of discussions, a lack of professional support during waiting periods, and re-explaining information to adult providers. Further, adolescents and caregivers expressed that successful transitions could be achieved through greater parental and provider involvement, as well as by implementing a ‘transition coordinator’. This would ideally be accompanied by a ‘transition passport’, giving adolescents instant access to their medical information, like their prescriptions, that could be shared with AMHS providers.

Wales et al. [45] explored the experience of transitioning, aiming to identify barriers and facilitators to this process. Providers were recruited via email from CAMHS and AMHS community ED teams, and asked to reflect on their past experiences. Four in-depth, semi-structured focus groups (1 h) were conducted, two in CAMHS (*n* = 10) and two in AMHS (*n* = 12). Additionally, ED patients (*n* = 29) who were eligible for transitions in the past two years, whether they transitioned or not, along with caregivers (*n* = 28), were recruited via letters from local CAMHS and AMHS. These individuals partook in in-depth, semi-structured qualitative interviews (50 min), either in person or over telephone. Thematic analysis revealed three core themes that served as barriers or facilitators to transitioning: (1) communication between CAMHS and AMHS internally and with patients and caregivers externally; (2) operational differences between CAMHS and AMHS, as well as uncertainty about transitions to AMHS; and (3) timing of transitions that are determined by rigid age boundaries and coincide with important life events.

Taking a resource perspective, one study [46] examined the perceived value of educational supports in facilitating effective transitions across ED care. Patients diagnosed with AN (*n* = 6), who transitioned to AMHS for at least one year, were recruited by administrative staff from a tertiary adolescent medicine program, supported by a dedicated ED team. In-depth, semi-structured qualitative interviews (30 min) were conducted via telephone. Thematic analysis identified three primary themes, namely unique challenges for ED patients (i.e., late age of ED onset and sudden discharge, fluctuating symptoms, and transition versus discharge), issues in adult care (i.e., greater autonomy and new psychiatric comorbidities), and the value and content of educational resources (i.e., as symptom benchmarks and provider connection tools). Specifically, patients thought educational supports would aid successful transitions into adult care, which should be distinguished from discharges, describe administrative changes, and set expectations for new roles and responsibilities.

Finally, Scanferla et al. [47] captured transition experiences shared by ED patients and their immediate family members. Recruited from an inpatient ED unit, 12 patients with AN and six related caregivers were included in the study. In-depth, semi-structured qualitative interviews were conducted in person for patients and over telephone for caregivers. Leveraging interpretative phenomenological analysis, an inductive approach that details how individuals make sense of their personal and social world [48], investigators revealed four main themes: (1) the transition experience in and of itself, comprising new caregiver roles, treatment modalities, and administrative cultures; (2) associated emotions, such as fear and abandonment (patients) and destabilization and helplessness (caregivers); (3) challenges of transitioning, like delayed access to services and lack of provider support; and (4) facilitators to improve the transition process, including smooth transfers, support of personal life goals, involvement of caregivers, and being accompanied and welcomed into adult care.

### Cross-sectional studies

**Fig. 3:**
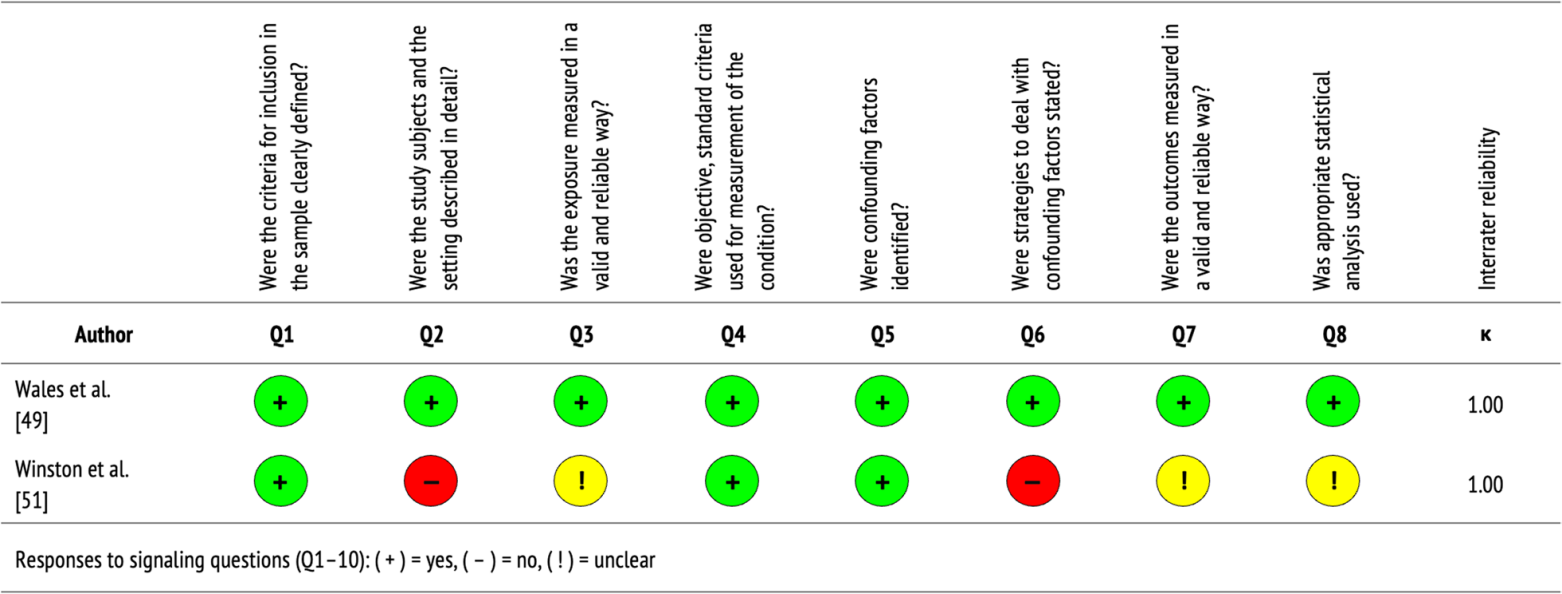
Quality assessment of cross-sectional studies

See Table 2 for a summary of the cross-sectional studies included in this review, which are further described below. Figure 3 displays the quality of evidence, specifically the extent to which each study addressed the possibility of bias in its design, conduct, and analysis. Overall, cross-sectional studies were of poor-to-moderate quality, with perfect agreement between independent reviewers who performed critical appraisals (Cohen’s *k* = 1.00).

**Table 2:** Characteristics of cross-sectional studies

Author	Objective	Location	Sample	Study design	Key findings
Wales et al. [49]	To assess the relative importance of qualitative statements about the transition from CAMHS to AMHS for EDs	BEAT, First Steps, Eating Disorders and Carers UK, United Kingdom	Patients, caregivers, providers (*n* = 28)	Prospective cross-sectional study, using a Q-methodology sort task, with a normal distribution pattern scale	Important factors: involving parents and caregivers in the transition process, facilitating effective transitions between services, supporting patients through transitions, and ensuring timely, patient-centered care
Winston et al. [51]	To establish how well recommendations for managing transitions from CAMHS to AMHS for EDs are being met	RCPsych National Training Days, England	Service teams (*n* = 38)	Prospective cross-sectional survey of CEDS-CYP teams, based on RCPsych guidance for ED transitions	Most teams compliant in providing transition protocols (52.6%), individual transition plans (78.9%), joint care with adult services (89.5%), and support to families (73.7%); yet few referred ED patients to specialist AMHS (15.8%)

*AMHS* adult mental health services, *CAMHS* child and adolescent mental health services, *CEDS-CYP* commissioning of local community eating disorders services for children and young people, *EDs* eating disorders, *RCPsych* Royal College of Psychiatrists

As an extension of their qualitative findings, Wales et al. [49] sought to determine which of the factors that influenced ED transitions were most important to medical stakeholders. Patients (*n* = 12), caregivers (*n* = 8), and providers (*n* = 8) were recruited from ED charities, support groups, and professional bodies. Eligible patients had been offered transitions from CAMHS to AMHS for an ED, whether they transitioned or not, and eligible providers had experience transitioning patients to AMHS, whether for an ED or an unrelated condition. Leveraging Q-methodology, a ‘qualiquantological’ approach that focuses on individual viewpoints and subsequently identifies shared ones [50], participants completed a Q-sort rank task, evaluating 40 qualitative statements based on their agreement. Principal component factor analysis identified four items, explaining 52% of the variance that highlighted: parent and caregiver inclusion, patient support during the transition, timely and patient-centered care, and effective transitions between services.

Most recently, a study [51] conducted in England assessed provider compliance with the National Health Services’ (NHS) recommendations for managing ED transitions for children and young people. During a series of national training days hosted by the Royal College of Psychiatrists, a survey was distributed to 70 teams that provided ED treatment. Of the 38 that participated, 97.4% (*n* = 37) had fixed transition boundaries, commonly set at 18 years of age. Moreover, 73.7% (*n* = 28) of teams reported that some young people were admitted to specialized or community-based programs for EDs or mental health issues, respectively, whereas 15.8% (*n* = 6) always transitioned to tertiary ED care. Further, 53.3% (*n* = 21) stated that a subset of ED patients did not meet clinical thresholds for AMHS, thereby failing to transition. Most teams complied with NHS recommendations, with 52.6% (*n* = 20) using ED-specific transition protocols, 78.9% (*n* = 30) creating individualized transition plans, 81.6% (*n* = 31) allowing flexible transition times, 89.5% (*n* = 34) jointly working with AMHS, and 73.7% (*n* = 28) providing transition support to caregivers and families. Regarding treatment, 97.4% (*n* = 37) of teams reported asymmetry between their therapeutic model and the services they transitioned care to, with more family-based approaches in CAMHS. Relatedly, 71.1% (*n* = 27) reported that providers discussed treatment differences with patients during the transition process, though 15.8% (*n* = 6) did not respond to this question.

### Longitudinal cohort studies

**Fig. 4:**
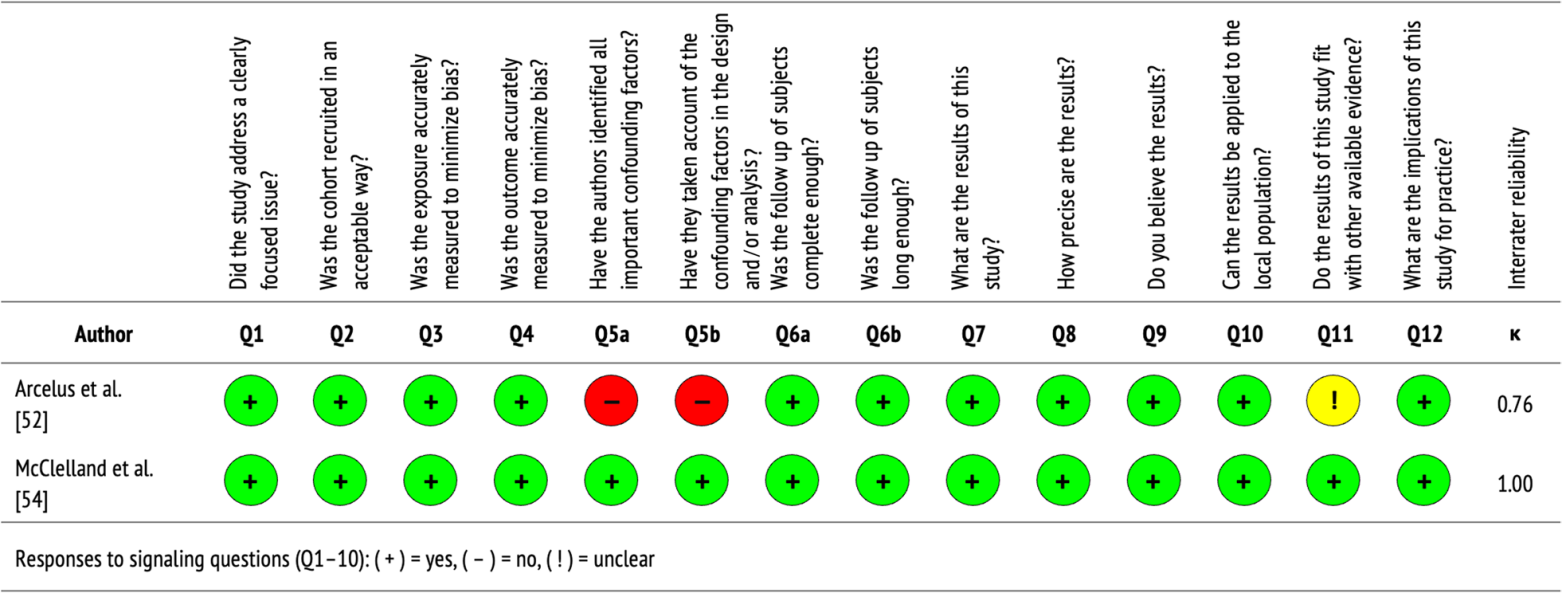
Quality assessment of longitudinal cohort studies

See Table 3 for a summary of the longitudinal cohort studies included in this review, which are further described below. Figure 4 displays the quality of evidence, specifically the extent to which each study addressed the possibility of bias in its design, conduct, and analysis. Overall, longitudinal cohort studies were of moderate-to-high quality, with almost perfect agreement between independent reviewers who performed critical appraisals (Cohen’s *k* = 0.88).

**Table 3:** Characteristics of longitudinal cohort studies

Author	Objective	Location	Sample	Study design	Key findings
Arcelus et al. [52]	To describe and compare young people in AMHS for EDs with and without past CAMHS contact; and to hypothesize differences	Leicester Adult Eating Disorder Service, NHS Trust, England	Patients (*n* = 206)	Retrospective medical chart review of young people (16–25) over 4-year period (2002–2005), using case records	Nearly half of patients referred to AMHS were referred by GPs versus CAMHS; those with past CAMHS contact, specifically as inpatients, presented with lower self-esteem and higher maturity fears than those without past contact
McClelland et al. [54]	To identify past CAEDS contact and predictors of future AMHS contact; and to delineate service use in adult services	MCCAED, SlaM NHS Foundation Trust, United Kingdom	Patients (*n* = 322)	Retrospective medical chart review of young adults (18–25) over 5-year period (2009–2014), using three databases	68.3% of patients with past CAEDS contact received no AMHS, 10.8% directly transitioned to AEDS, and 7.6% were re-referred to AEDS after CAEDS discharge; older age and increased CAEDS contact predicted increased AEDS contact

*AMHS* adult mental health services, *CAMHS* child and adolescent mental health services, *EDs* eating disorders, GPs general practitioners, *MCCAED* Maudsley Centre for Child and Adolescent Eating Disorders, *NHS* National Health Service, SlaM South London and Maudsley

Arcelus et al. [52] were the first to compare new ED patients in AMHS to those with prior contact in CAMHS, examining differences in illness severity and complexity. Referrals to AMHS were made from local primary care and secondary psychiatric services. Through a medical chart review, 206 young people (aged 16–25) were identified over a four-year period (2002–2005). Results showed that 27.7% (*n* = 57)  were previously treated in CAMHS for an ED, either as in-patients (57.9% [*n* = 33]) or out-patients (42.1% [*n* = 24]). Surprisingly, 43.9% (*n* = 25) were referred to AMHS by general practitioners versus CAMHS. Further, patients with prior CAMHS contact, particularly as in-patients, had lower self-esteem and higher maturity fears than those without; the latter referring to a desire to return to childhood, or a fear of adulthood and its many demands [53].

To further characterize service utilization, another study [54] analyzed a consecutive cohort of patients (aged 13–17) treated in CAMHS for an ED over a five-year period (2009–2014). Data regarding their use of AMHS for ED care as young adults (aged 18–25), within a follow-up period (2013–2017), were extracted from local and national hospital records. A total of 322 patients were identified and eligible for inclusion. Of these patients, 67.0% (*n* = 216) made no contact with AMHS, 13.0% (*n* = 42) received brief ED treatment, 10.0% (*n* = 32) received extended ED treatment, and 10.0% (*n* = 32) received non-ED treatment. Moreover, 10.8% of patients were directly referred to AMHS, whereas 7.6% were re-referred by CAMHS back to their general practitioner following discharge. Older age at presentation to CAMHS predicted increased ED care in young adulthood, as did the amount of CAMHS service use (i.e., the number and length of out-patient, day-patient, and/or in-patient episodes). No/brief use of AMHS was associated with the longest duration of untreated EDs.

## Discussion

Based on the present review, transitions from CAMHS to AMHS for EDs are complex, multifaceted, and challenging for patients, caregivers, and providers alike. The 18th birthday often signals a change in (or loss of) ED services, despite nearly a quarter of patients requiring ongoing, specialty care [54], whether due to early ED onset or illness chronicity. This disruption likely aggravates cognitive and behavioral symptoms, as well as the risk of functional disability and premature death [1, 55]. Moreover, the transition period is a time of newly acquired autonomy, legal power, and responsibility, which patients and caregivers may not be ready for. Developmental delays and ambivalence towards recovery [17], for instance, can negatively impact patients’ motivation for and readiness to transition. Additionally, the ‘duty of confidentiality’ may preclude caregivers’ access to their child’s medical records without their explicit consent. This can lead to caregivers feeling helpless and psychologically distressed [43]. On a systemic level, asymmetry between services may interfere with effective transitions [45], resulting in administrative mistrust and poor transfer alliance [40]. Providers may also lack competency in transitioning ED patients to AMHS for their condition [40, 43], and may develop professional insecurity as a byproduct, with negative downstream effects on patients and caregivers [39, 40, 47].

**Fig. 5:**
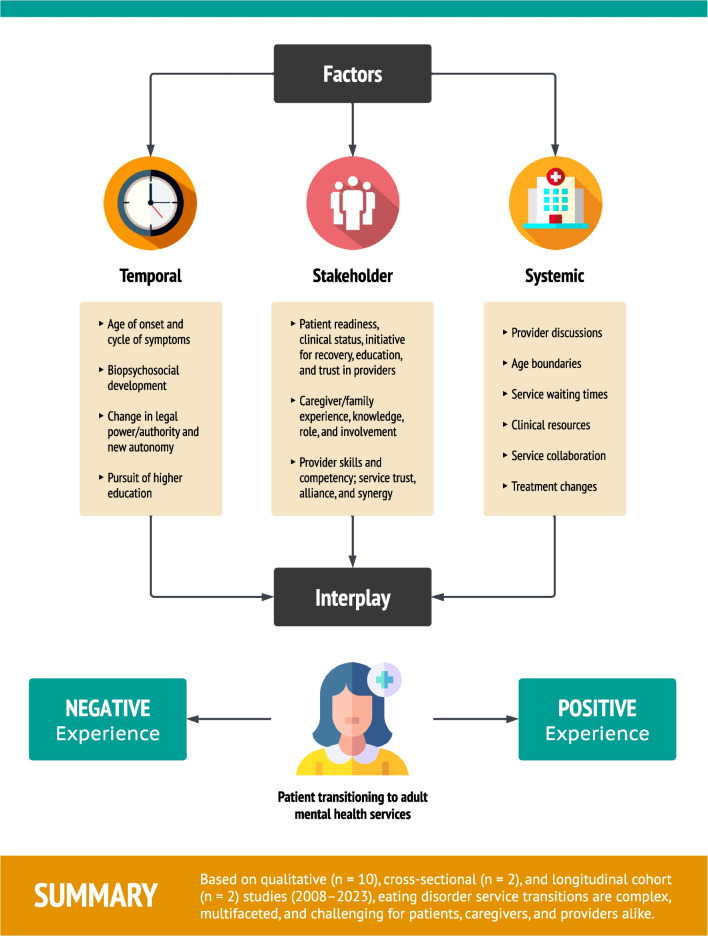
Factors impacting transitions

Overall, the prospect of smooth and effective transitions appears low, stemming from an interplay of temporal-, stakeholder-, and systemic-related factors (Fig. 5). However, these findings are largely predicated on qualitative studies that dominate 71% of the literature. Only two cross-sectional studies examined transitions between CAMHS and AMHS, neither of which assessed patient outcomes. Longitudinal cohort studies were additionally limited by number and research design, primarily investigating service utilization, with only one study measuring ED symptoms post-transition, using validated psychometric scales. This highlights critical knowledge gaps. For example, it is unclear whether transitions are problematic in the longer term, or a necessary and positive step towards recovery: “While some [patients] described the transition to AMHS as something that prolonged their recovery process, others described it as vital for their improvement.” [42] Nonetheless, the field stands to benefit from a framework that addresses key factors shown to impact transitions across  ED care, which can supplement or be integrated into ‘good practice’ guidelines for managing transitions. In what follows, we propose an actionable, evidence-based framework (TRIPS) that comprises five transition strategies, designed to provide necessary guidance and support for all stakeholders affected by this service change—patients, caregivers, and providers. The hope is to alleviate challenges during this period and subsequently improve clinical trajectories.

## TRIPS framework

### Timely talks

Providers are encouraged to initiate early discussions with patients and caregivers about the transition to AMHS [44, 49], ideally starting at age 14 and concluding by 18, as suggested by evidence on health transitions, broadly [56]. They should aim for a minimum of three transition-related discussions over this period, which can complement regular patient visits and be conducted in-person or via teleconferencing, such as Zoom or Skype [45]. This can help build trusting relationships [42], mutual understanding [44], and motivation for treatment [42], while cognitively and gradually phasing patients out of CAMHS and preparing them for AMHS [44].

### Readiness

Providers are encouraged to evaluate patients’ and caregivers’ readiness for transitioning [38, 40, 42], which can be conducted through early discussions. Readiness assessments should consider patients’ developmental stage and progress (e.g., autonomy and social connections) [17, 42–44], important life events and ambitions (e.g., leaving home and pursuing higher education) [39, 45], and initiative for recovery (e.g., uncertainty and hopefulness) [17, 40, 41]. Providers should also evaluate patients’ and caregivers’ responsibilities [17, 39, 40, 43, 44], specifically their current roles and obligations, and how these might change during the transition process. Critically, providers should assess whether patients meet threshold criteria for AMHS [45], which can help avoid unnecessary waiting periods and unsuccessful referrals.

### Inclusion

Providers are encouraged to include caregivers in transition discussions and related decisions [17, 40, 43, 44, 47, 49], as deemed appropriate. They should be cognizant of caregivers’ knowledge and understanding of the nature and management of their child’s ED [43], and how that insight can be positively leveraged in the transition process. Sharing medical information with caregivers, such as their child’s test results, treatment objectives, and clinical progress, may ease potential psychological distress for both stakeholders [43]. It is particularly central to share this information before patients reach their transition boundary or discharge from CAMHS, when privacy laws and legal responsibility will shift. Moreover, providers should offer recommendations for continued care and referrals to providers in AMHS, as able [39, 44].

### Preparation

Providers are encouraged to direct patients and caregivers to resources that can prepare them for  AMHS [47]. This is critical during waiting periods [43, 44] when patients likely depend on mental health services [42], yet none are provided, as this may otherwise increase the risk for ED relapse [43]. Patients may benefit from educational tools that explain the transition process and detail their personal information [46], as well as interventions that target psychological and developmental changes [38], integrate self-management skills [38], promote autonomy, independence, and self-sufficiency [17, 38, 39, 42], and facilitate personal life goals [47]. Caregivers may also benefit from psychoeducation [38, 39] and mental health services [38, 43], specifically information on treatment differences between CAMHS and AMHS [39], and psychotherapy that gradually decreases their role as ‘managers’ and increases their role as ‘supporters’ [39]. Individuals with lived experiences with EDs, often accessible through support groups, can additionally serve as resources for patients and caregivers [45].

### Synergy

Providers are encouraged to facilitate effective transitions by communicating [39, 45] and collaborating [39, 49] between services and providers. They should be aware of administrative, cultural, and ideological differences between CAMHS and AMHS [40, 45], otherwise known as ‘organizational boundaries’, regarding treatment values, approaches, and procedures, and should coordinate patient care to the best of their ability [38, 43]. If an AMHS provider has been identified, providers should set up an introductory meeting with them, alongside patients and caregivers, to facilitate mutual understanding, trust, and synergy amongst all stakeholders [40]. This can also help prevent patients and caregivers from re-explaining medical information to AMHS providers [44].

## Looking ahead

In summary, the TRIPS framework may serve as a valuable and wide-reaching transition tool for ED patients, caregivers, and providers, and may inform the development of other resources, such as a ‘transition passport’, as suggested by one study [44] in this review. While transition passports have yet to be implemented for EDs, to the best of our knowledge, they have been used for other medical disorders. For example, Dwyer et al., [57] iteratively developed and tested a digital ‘mobile health transition passport’ for Klinefelter syndrome, a chronic genetic condition where young adults who are lost to ‘service gaps’ face significant sequelae in their health and well-being [58]. The transition passport was designed to educate patients about their illness, promote self-management skills, and support continuity of care. Based on patient support groups, Dwyer et al. produced a transition passport that was functional, understandable, empowering, and easily actionable [57]. It was also highly practical for patients and integrated well into existing healthcare workflows. Transition passports can additionaly standardize information that is transferred between providers across services, which has been problematic in ED care [44]. As shown in a recent systematic review on health systems [59], digital personal health records, like transition passports, significantly improve the quality of treatment and healthcare delivery and promote engagement between patients, providers, and services. They also present a relatively low-cost, scalable solution for supporting patients with medical comorbidity and serious mental illnesses [54]. Hence, the TRIPS framework and transition passports may be worthwhile future considerations for improving transition experiences and service collaboration among ED stakeholders.

## Limitations

This review and the generalizability of its findings is inherently limited. This is due to an under-developed literature that comprises only 10 qualitative, two cross-sectional, and two longitudinal cohort studies. For qualitative studies, the majority stemmed from two specialist ED programs in Canada and Norway, which focused on AN over other ED types. For cross-sectional studies, both originated from the United Kingdom and had underpowered sample sizes. For longitudinal cohort studies, both were conducted in the United Kingdom and used varying methods and cutoff points for assessing transitions and related outcomes. Future research should incorporate valid measures and refine study parameters, such as the duration between last CAMHS contact and present AMHS contact (e.g., 12 months) to further characterize service pathways. This will aid more quantitative approaches, including meta-analyses, to precisely estimate transition effects both acutely and over time. Larger, more inclusive and diverse studies are also warranted.

## Conclusion

Across healthcare systems internationally, transitions from CAMHS to AMHS for young people with EDs appear problematic. Despite this, transitions may be a necessary part of personal development and mental health recovery. We propose the actionable, evidence-based framework, TRIPS (Timely talks, Readiness, Inclusion, Preparation, and Synergy), which comprises five transition strategies designed to facilitate effective transfers across ED care. The TRIPS framework aims to support transition experiences, alleviate challenges during this time, and subsequently improve clinical trajectories.

### Supplementary Information

**Additional file 1**. **Table S1**: Search strategy. **Table S2**: PICOS criteria.

## Data Availability

The datasets used and/or analyzed during the current study are available from the corresponding author (A.R.) upon reasonable request.

## Abbreviations

AMHS: Adult mental health services
AN: Anorexia nervosa
BED: Binge eating disorder
BN: Bulimia nervosa
CAMHS: Child and adolescent mental health services
ED: Eating disorders
NHS: National Health Services
PICOS: Population, intervention, comparison, outcomes, and study design
PRISMA: Preferred reporting items for systematic reviews and meta-analyses
TRIPS: Timely talks, readiness, inclusion, preparation, and synergy
