# Correction: Transitions from child and adolescent to adult mental health services for eating disorders: an in-depth systematic review and development of a transition framework

**DOI:** 10.1186/s40337-024-01022-y

**Published:** 2024-06-03

**Authors:** Anya Ragnhildstveit, Nandita Tuteja, Paul Seli, Leo Smart, Naz Uzun, Lisa C. Bass, Alyssa C. Miranda, Tamsin J. Ford, Sharon A. S. Neufeld

**Affiliations:** 1 Department of Psychiatry, University of Cambridge, Cambridge, England, UK; 2 Department of Neurobiology and Behavior, University of California, Irvine, Irvine, CA USA; 3 Department of Psychology and Neuroscience, Duke University, Durham, NC USA; 4 Neuroscience Program, Bates College, Lewiston, ME USA; 5 Department of Psychology, University of Manchester, Manchester, England, UK; 6 Neuroscience Interdepartmental Program, University of California, Los Angeles, Los Angeles, CA USA; 7 Consciousness and Transformative Studies, National University, San Diego, CA USA

**Correction to: Journal of Eating Disorders (2024) 12:36**

10.1186/s40337-024-00984-3

Due to typesetting error, the original article contained an incorrect version of Fig. 1, as follows:


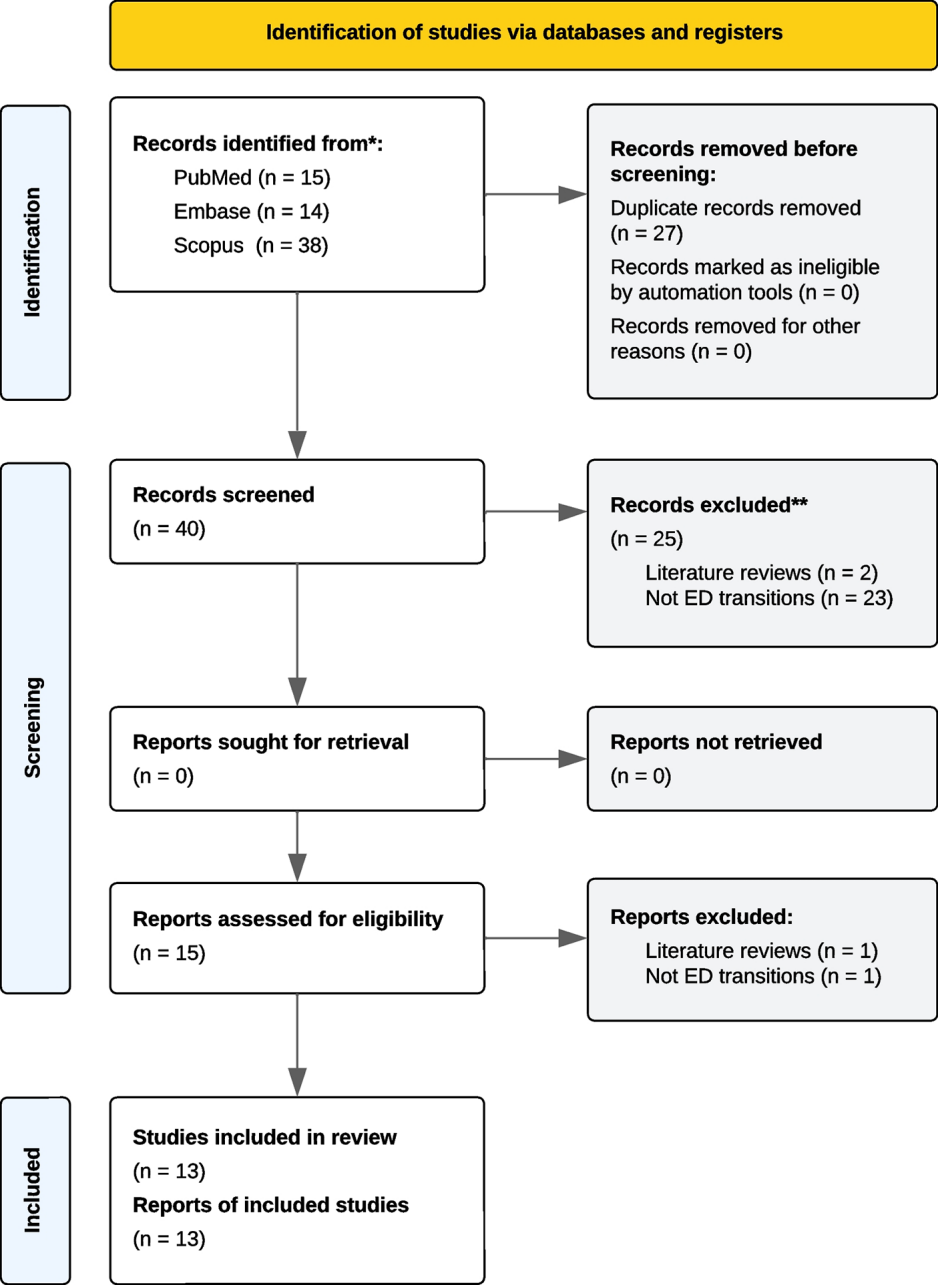


The correct Fig. 1 is as follows:


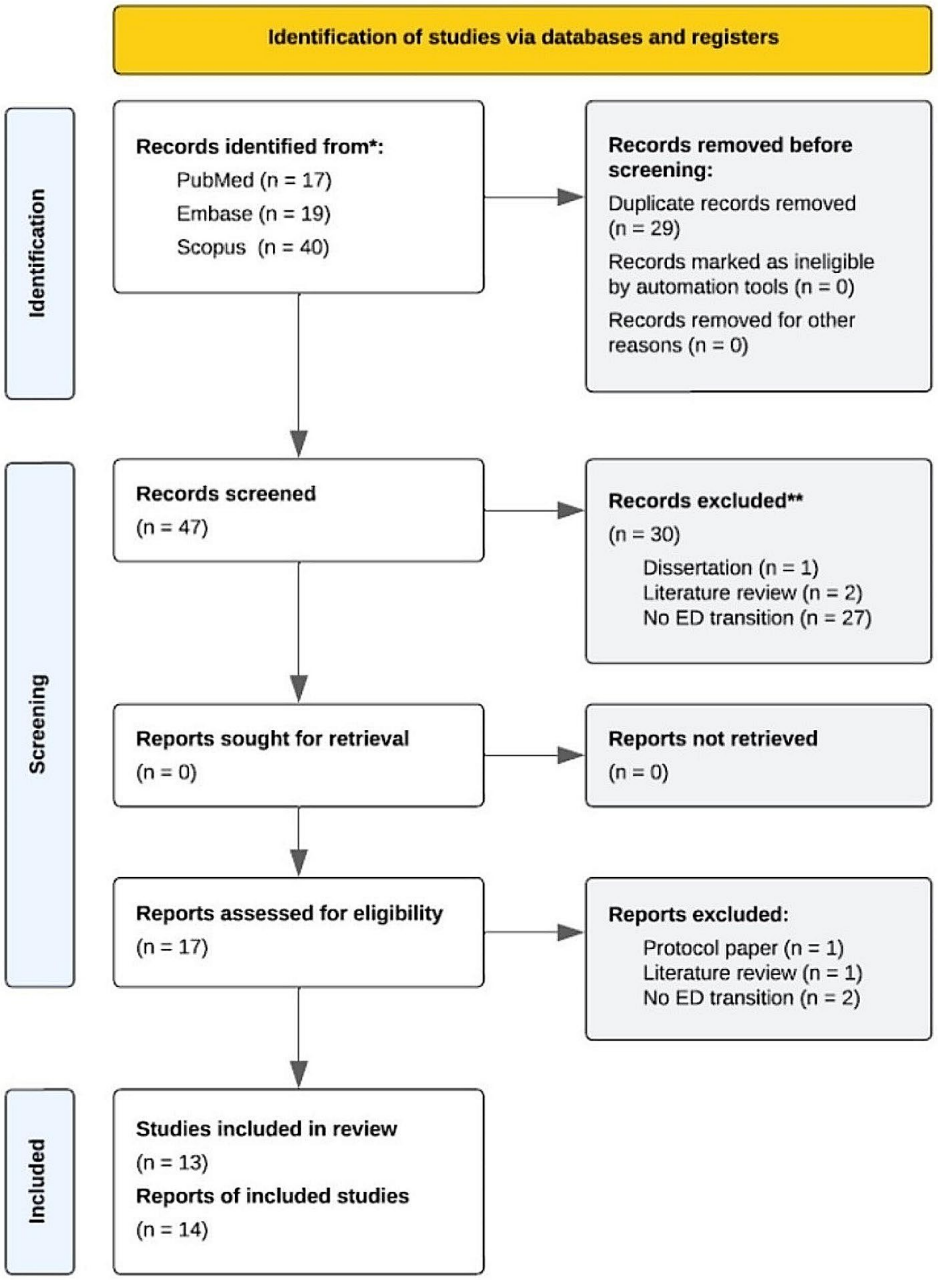


The original article has been corrected.

