# Supplementary material for: Transitions from child and adolescent to adult mental health services for eating disorders: an in-depth systematic review and development of a transition framework
Source: J Eat Disord. 2024 Mar 7;12:36. doi: 10.1186/s40337-024-00984-3 (PMC10921655; doi:10.1186/s40337-024-00984-3)
Supplement: Supplementary file 1 — Additional file 1. Table S1: Search strategy. Table S2: PICOS criteria. [file 40337_2024_984_MOESM1_ESM.docx]

**Supplementary Material**

**Table S1.** Search strategy.

| **Database** | **Results** | **Search String** |
| --- | --- | --- |
| **PubMed** | 17 Articles | ("eating disorder"[Title] OR "anorexia"[Title] OR "anorexia nervosa"[Title] OR "bulimia"[Title] OR "bulimia nervosa"[Title] OR "binge eating"[Title] OR "binge eating disorder"[Title] OR "orthorexia"[Title] OR "other specified feeding or eating disorder"[Title] OR "avoidant restrictive food intake disorder"[Title] OR "pica"[Title] OR "rumination disorder"[Title]) AND ("transition"[Title] OR "transitioning"[Title] OR "transfer"[Title] OR "transferring"[Title] OR "across"[Title] OR "crossing"[Title]) AND ("service"[Title] OR "services"[Title] OR "treatment"[Title] OR "treatments"[Title] OR "program"[Title] OR "programs"[Title] OR "care"[Title]) AND ("humans"[MeSH Terms] AND "english"[Language]) |
| **Embase** | 19 Articles | ('eating disorder':ti OR 'anorexia':ti OR 'anorexia nervosa':ti OR 'bulimia':ti OR 'bulimia nervosa':ti OR 'binge eating':ti OR 'binge eating disorder':ti OR 'orthorexia':ti OR 'other specified feeding or eating disorder':ti OR 'avoidant restrictive food intake disorder':ti OR 'pica':ti OR 'rumination disorder':ti) AND ('transition':ti OR 'transitioning':ti OR 'transfer':ti OR 'transferring':ti OR 'across':ti OR 'crossing':ti) AND ('service':ti OR 'services':ti OR 'treatment':ti OR 'treatments':ti OR 'program':ti OR 'programs':ti OR 'care':ti) AND [article]/lim AND [humans]/lim AND [english]/lim AND [embase]/lim |
| **Scopus** | 40 Articles | TITLE ( ( "eating disorder" OR anorexia OR "anorexia nervosa" OR bulimia OR "bulimia nervosa" OR "binge eating" OR "binge eating disorder" OR orthorexia OR "other specified feeding or eating disorder" OR "avoidant restrictive food intake disorder" OR pica OR "rumination disorder" ) AND ( transition OR transitioning OR transfer OR transferring OR across OR crossing ) AND ( service OR services OR treatment OR treatments OR program OR programs OR care ) ) AND ( LIMIT-TO ( SRCTYPE , "j" ) ) AND ( LIMIT-TO ( PUBSTAGE , "final" ) ) AND ( LIMIT-TO ( DOCTYPE , "ar" ) ) AND ( LIMIT-TO ( LANGUAGE , "English" ) ) |
| NOTE. Electronic databases were queried from inception to December 3, 2023. | | |

**Table S2.** PICOS criteria.

| **Criteria** | **Inclusion Criteria** | **Exclusion Criteria** |
| --- | --- | --- |
| **Population** | Young people (aged 15–25) diagnosed with an ED of any type, severity, and duration; caregivers of young people diagnosed with an ED; or providers treating young people diagnosed with an ED. | Infants, children, or adolescents (aged < 15) and young people (aged 15–25) not diagnosed with an ED. |
| **Intervention** | Transitions from CAMHS to AMHS for EDs, whether impending or completed, in tertiary or community care settings. | Transitions from CAMHS to AMHS for non-ED diagnoses. |
| **Comparison** | Not limited to comparator studies, including studies with no control. | Not applicable. |
| **Outcome** | Experiential (attitudes, perspectives, or experiences) or clinical (symptoms, health, function, quality of life, or survival) outcomes related to any step of the transition pathway, including referral, assessment, treatment, or discharge. | Experiential or clinical outcomes not related to any step of the transition pathway. |
| **Study Design** | Qualitative, quantitative, or mixed methods studies, whether retrospective or prospective, peer-reviewed and published in English. | Case studies, reviews, editorials, expert opinion pieces, commentaries, letters to the editor, and articles with inaccessible full texts; studies not peer-reviewed or not published in the English. |
| AMHS, adult mental health services; CAMHS, child and adolescent mental health services; EDs, eating disorders | | |
